# Calcium Signaling Is Impaired in PTEN-Deficient T Cell Acute Lymphoblastic Leukemia

**DOI:** 10.3389/fimmu.2022.797244

**Published:** 2022-02-02

**Authors:** Saran Pankaew, Delphine Potier, Clémence Grosjean, Mathis Nozais, Julie Quessada, Marie Loosveld, Élisabeth Remy, Dominique Payet-Bornet

**Affiliations:** ^1^ Aix Marseille Univ, CNRS, INSERM, CIML, Marseille, France; ^2^ Aix Marseille Univ, CNRS, I2M, Marseille, France; ^3^ APHM, Hôpital La Timone, Laboratoire d’Hématologie, Marseille, France

**Keywords:** thymocytes, T-ALL, PTEN, TCR signaling, calcium signaling, single-cell RNA-seq, qualitative mathematical model

## Abstract

PTEN (Phosphatase and TENsin homolog) is a well-known tumor suppressor involved in numerous types of cancer, including T-cell acute lymphoblastic leukemia (T-ALL). In human, loss-of-function mutations of *PTEN* are correlated to mature T-ALL expressing a T-cell receptor (TCR) at their cell surface. In accordance with human T-ALL, inactivation of *Pten* gene in mouse thymocytes induces TCRαβ^+^ T-ALL development. Herein, we explored the functional interaction between TCRαβ signaling and PTEN. First, we performed single-cell RNA sequencing (scRNAseq) of PTEN-deficient and PTEN-proficient thymocytes. Bioinformatic analysis of our scRNAseq data showed that pathological Pten^del^ thymocytes express, as expected, *Myc* transcript, whereas inference of pathway activity revealed that these Pten^del^ thymocytes display a lower calcium pathway activity score compared to their physiological counterparts. We confirmed this result using *ex vivo* calcium flux assay and showed that upon TCR activation tumor Pten^del^ blasts were unable to release calcium ions (Ca^2+^) from the endoplasmic reticulum to the cytosol. In order to understand such phenomena, we constructed a mathematical model centered on the mechanisms controlling the calcium flux, integrating TCR signal strength and PTEN interactions. This qualitative model displays a dynamical behavior coherent with the dynamics reported in the literature, it also predicts that PTEN affects positively IP3 (inositol 1,4,5-trisphosphate) receptors (ITPR). Hence, we analyzed *Itpr* expression and unraveled that ITPR proteins levels are reduced in PTEN-deficient tumor cells compared to physiological and leukemic PTEN-proficient cells. However, calcium flux and ITPR proteins expression are not defective in non-leukemic PTEN-deficient T cells indicating that beyond PTEN loss an additional alteration is required. Altogether, our study shows that ITPR/Calcium flux is a part of the oncogenic landscape shaped by PTEN loss and pinpoints a putative role of PTEN in the regulation of ITPR proteins in thymocytes, which remains to be characterized.

## Introduction

T-cell acute lymphoblastic leukemia (T-ALL) is a malignant proliferation of T cell progenitors. T-ALL represents 10-15% of pediatric and 20-25% of adult cases of ALL (1). This disease is a result from multiple genetic alterations affecting oncogenes and tumor suppressor genes. Among them, loss-of-function mutations of PTEN (Phosphatase and TENsin homolog) are recurrently observed (2). PTEN is a phosphatase which dephosphorylates phosphatidylinositol (3–5)-triphosphate (PIP3) into PIP2. Thus, PTEN antagonizes the function of Phosphatidylinositol 3 kinase (PI3K) and is the main negative regulator of PI3K/AKT signaling pathway. *PTEN* gene is a well-known tumor suppressor involved in numerous types of cancer (3). In T-ALL, PTEN inactivation is correlated to mature T-ALL expressing a T-cell receptor (TCR) at their cell surface and is associated with poor prognosis (4, 5). In accordance with human T-ALL, the deletion of *Pten* gene in mouse thymocytes induces TCRαβ^+^ T-ALL development (6–8).

Based on CD4 and CD8 expression, the successive steps of thymic maturation of αβ T cells are roughly composed of CD4^-^CD8^-^ double negative (DN), immature CD8 single-positive (ISP), CD4^+^CD8^+^ double-positive (DP) and finally CD4 or CD8 single-positive (SP) stages. The ‘raison d’être’ of a thymocyte is to express a functional TCR that is able to induce an immune response against a foreign antigen, while avoiding self-antigenic reaction. Therefore, quality controls of the TCR, namely positive and negative selections, occur during thymopoiesis. The positive selection aims to select thymocytes expressing a ‘fit’ TCR, which interacts with medium affinity for self-peptide-major histocompatibility complex (p-MHC). In contrast, thymocytes bearing an ‘unfit’ TCR that has no or too high affinity for p-MHC are eliminated by death-by-neglect or negative selection, respectively (9, 10). Consequently, in a physiological setting, TCR signaling dictates thymocyte fate. In a previous report (11), we investigated the impact of TCRαβ signaling during leukemogenesis. We showed that a fit TCRαβ signaling prevents T-ALL development of PTEN-deficient thymocytes while thymocytes harboring unfit TCR (that should have been eliminated during positive selection) are selected for leukemogenesis.

In this work, we seek to understand the functional interaction between TCRαβ signaling and PTEN. For this purpose, we used *Pten^flox/flox^* conditional mouse model (12) crossed with transgenic CD4-Cre mice. In the resulting Pten^del^ mice, *Pten* gene is inactivated during thymopoiesis and that leads, as stated above, to TCRαβ^+^ T-ALL development (6–8). First, we performed single-cell RNA sequencing (scRNAseq) assays whose analysis suggests that in Pten^del^ tumor cells the calcium signaling is impacted.

We confirmed these results using *ex vivo* assays and explore the mechanism of calcium pathway alteration through the construction of a mathematical model. Collectively our data uncovered that the level of IP3 receptors is strongly reduced in leukemic PTEN-deficient cells and we further found that the TCR-induced Ca^2+^ flux is abolished in these leukemic cells.

## Materials and Methods

### Mice

Mice were bred and housed in specific pathogen-free conditions in CIML animal facilities and were handled in accordance with French and European guidelines. Procedures of the project have been validated by French ethical committee (project APAFIS#4484-2016031113534101). Conditional Pten^flox/flox^ mice (12) were obtained from European Mouse Mutant Archives (EMMA). CD4-Cre mice (13) and OT-II mice (14) were bred and maintained in CIML animal facilities. Immunodeficient NOD.Cg-*Prkdc^scid^II2g^tm1Wji^*/SzJ mice (abbreviated NSG) are from Charles River. Leukemic Pten^del^ mice were usually analyzed between 10 and 15 weeks of age, while pre-leukemic Pten^del^ mice were usually aged between 4 and 8 weeks.

### Flow Cytometry Analysis

Single-cell suspensions were stained with conjugated antibodies for 30 min at 4°C and washed twice with FACS buffer (PBS, 2% fetal bovine serum (FBS), 1 mM EDTA). Multicolor flow cytometry analysis was performed with FACS Canto II (Becton–Dickinson) and data analyzed with FlowJo software (FlowJo, Becton–Dickinson). Antibodies used for flow cytometry are listed in **Supplementary Table 1**.

### Single-Cell RNA Sequencing Experiment

Thymus and spleens were processed as described previously (15). Briefly, they were dilacerated and treated with 1 mL RBC Lysis Buffer (ThermoFischer Scientific). Then cells were resuspended in PBS supplemented with 2% FBS. Splenic T cells were further purified using EasySep™ Mouse T Cell Isolation Kit (StemCell technologies). Each sample was labelled with a distinct TotalSeq™-A anti-mouse Hashtag reagent (BioLegend). The different samples were pooled, then loaded on a Chromium Chip B (10X Genomics), and cells were droplet-encapsulated with a Chromium Controller (10X Genomics). Single-cell cDNA synthesis and sequencing libraries were prepared with Chromium Single Cell 3’ v3 Library and Gel Bead kit (10X Genomics) according to manufacturer’s instructions. Libraries were sequenced using a Next-seq500 (Illumina) and the following parameters, Read1: 26 cycles, i7: 8 cycles, Read2: 57 cycles.

### Single-Cell RNA Sequencing Data Analysis

For data preprocessing, mRNA library reads were aligned to mm10 mouse genome (GRCm38) and quantified using CellRanger count (version 3.0.1). Hashtag oligos counts for cell hashing were quantified using CITE-seq-count version 1.4.3 (16), with default parameters. The produced mRNA and HTO data matrices were imported into R (v 4.0.3) and downstream analysis were performed with the Seurat package version 4.0.0 (17, 18).

To perform sample demultiplexing, hashtag oligos (HTOs) counts for each cell were normalized using a centered log ratio (CLR) transformation across cells. Then, cells were demultiplexed using the Seurat MULTIseqDemux function. Doublets and background empty droplets (negative) were subsequently removed. Before mRNA expression analysis, low quality cells were filtered from the mRNA matrix (cells expressing < 200 genes, gene expressed in < 3 cells, or cells with a proportion of mitochondrial transcripts > 10%). The expression raw counts were normalized using NormalizeData function in Seurat with the default parameters. Top 2000 highly variable features were selected using ‘FindVariableFeatures’ function in Seurat. The prediction of cell cycle phase was performed using the CellCycleScoring Seurat function, which attributes S and G2/M scores and predicts classification of each cell in either G2/M, S or G1 phase. Normalized expression data were scaled and regressed out for cell cycle variation using Seurat ‘ScaleData’ function with S and G2/M scores.

We performed a principal component analysis (PCA) using ‘RunPCA’ function on the scaled data (npc = 50) and the top 25 principal components (PCs) were selected for the downstream non-linear dimension reduction and clustering. A ‘Uniform Manifold Approximation and Projection’ (UMAP) was performed using the ‘RunUMAP’ function to embed cells in 2-dimensions space. Cell clusters were identified using Seurat ‘FindNeighbors’ and ‘FindClusters’ (using Louvain Algorithm option and a resolution of 1.5) functions. Non-T cells cluster and cluster containing only Tγδ cells were excluded from the downstream analysis. After the dataset cleaning, top 2000 highly variable features were re-selected and scaled as previously described. PCA and UMAP were re-calculated using 20 PCs. The final cell clusters were identified using Seurat ‘FindNeighbors’ and ‘FindClusters’ (with Louvain Algorithm option) functions with the first 20 PCs and coarse grain resolution of 0.7. Each computed cell clusters were assigned to thymopoiesis stages by manual curation, using thymopoiesis stage markers previously identified (19–22) and Immgen Database (23). To infer pathway activity, genes sets of pathways of interest (**Supplementary Table 2**) were retrieved from Molecular Signature Database (MSigDB) (24) and were used to score pathway activity for each cell with AUCell version 1.12.0 (25).

### Logical Modelling

The logical modelling is a qualitative approach used to represent and analyze gene regulatory networks (26, 27). It relies on a Regulatory Graph (RG) that is a directed signed graph whose nodes represent the biological components and edges, the regulation between them (positive edges for activations, and negative for inhibitions). At each component is associated a discrete variable representing its qualitative level of activity, called its state, and a logical rule, expressed with AND, OR, and NOT logic gates, specifying the dynamics for each node depending on the state of its regulators (nodes that are sources of an incoming edge). We hence parameterize the model with a set of logical rules that define the global dynamics of the system. Simulations of the logical model are performed in the asynchronous updating scheme, providing the possible trajectories of the system, i.e., sequence of successive states guided by the logical rules. As the number of states is finite, simulations will eventually lead to attractors, that can be categorized into two types: single attractors (fixed stable states), or cyclic attractors, containing at least two states. The logical model of TCR-induced calcium flux was constructed, fed with current knowledge from the literature and analyzed using GINsim version 3.0 software (28). The references for each component and interaction were annotated in the .ginml file.

### Transplantation of Leukemic Cells From *Cdkn2a^-/-^* T-ALL Mouse Model

The Cdkn2a*^-/-^* T-ALL mouse model is described in Gon et al. (11): briefly, bone marrow cells from *Cdkn2a^-/-^* mice were harvested and co-cultured for 10 days on OP9-DL1 stromal cells. Then cells were injected into NSG mice in order to generate T-ALL in around 4 months. For our study, around 1×10^6^ cells from three distinct *Cdkn2a^-/-^* T-ALL (11) were intravenously injected into tail vein of immunodeficient NSG mice. At first signs of disease (around 3 weeks post-transplantation), *Cdkn2a^-/-^* grafts were harvested from the spleen of NSG mice and were directly used for calcium flux assays.

### Calcium Flux Assay

Thymus and/or spleens from Control, Pten^del^ or *Cdkn2a^-/-^* mouse models, were dilacerated on a 70 µM nylon mesh (Corning) and treated with 1 mL RBC Lysis Buffer (ThermoFischer Scientific). Then, total thymocytes or splenocytes were washed in complete HBSS (HBSS supplemented with 0,1% bovine serum albumin, 1 mM MgCl_2_, 1 mM CaCl_2_, 10 mM HEPES buffer), loaded with Indo-1 AM (10 µM final) and incubated 20 min at 37°C with 5% CO_2_. After washing in complete HBSS, cells were stained 10 min at room temperature (RT) with anti-CD4-APC and anti-CD8-PE antibodies. Following staining, cells were washed and resuspended at 50×10^6^ cells/mL in complete HBSS. 5×10^6^ cells were first incubated with 5 µg of biotinylated anti-CD3 antibodies during 1 min at RT, then following the addition of 400 µL of warm (37°C) complete HBSS, cells were analyzed using a LSRII instrument (Becton Dickinson). We recorded during 10 min the calcium flux that was measured as the ratio of fluorescence emission at 410 nm (bound Indo-1) *versus* 475 nm (free Indo-1). Baseline fluorescence of anti-CD3-bound cells was monitored for 30 seconds before the addition of streptavidin (20 μg/mL final) to induce TCR cross linking and stimulation. Then 2 µL of ionomycin (stock solution at 1 mg/mL) was added at 8 min. Addition of ionomycin elicited an immediate Ca^2+^ response in all samples. FACS data were analyzed with FlowJo software (FlowJo, Becton–Dickinson).

### Immunoblotting

Around 2×10^7^ cells from total thymus (or from total spleen for *Cdkn2a* ^-/-^ mouse model) were lysed for 15 min at 4°C in lysis buffer (50 mM Tris-HCl pH 8, 0.02% Nonidet P-40, 20 mM EDTA pH 8) supplemented with protease and phosphatase inhibitors cocktail tablets (Roche). Then protein extracts (~80 μg) were separated by SDS-PAGE and transferred to nitrocellulose membrane using Iblot Gel Transfer stacks and Iblot system (Invitrogen). Membranes were blocked in TTBS (137 mM NaCl, 2 mM KCl, 25 mM Tris and 0.1% Tween 20) supplemented with 5% bovine serum albumin (BSA) and incubated with primary antibodies. The fluorescent secondary antibodies were added for 1 hour at room temperature. Antibodies used for immunoblotting are listed in **Supplementary Table 3**. Immunoblots were analyzed using an Odyssey^®^ infrared imaging system (Li-Cor Biosciences). For multiple probing, blots were stripped using Restore western blot stripping buffer (Pierce). Densitometric analysis were performed using ImageJ software.

### TCR Stimulation Assays

For TCR stimulation assays 2×10^7^ cells from total thymus (or from total spleen for *Cdkn2a* ^-/-^ mouse model) were resuspended in RPMI medium and first dispatched in two tubes (1×10^7^ cells each): unstimulated and stimulated. In the stimulated samples, biotinylated anti-CD3 (10 μg; clone 2C11, BD Pharmingen) and biotinylated anti-CD28 (10 μg; clone 37.51, BD Pharmingen) were added and the samples were incubated 5 min at 37°C. Then 14 μg of streptavidin (Thermo Scientific) were added in both stimulated and unstimulated samples that were further incubated 2 min at 37°C. Finally cells were lysed by the addition (vol/vol) of 2X lysis buffer (100 mM Tris, 2% Nonidet P-40, 40 mM EDTA) supplemented with protease and phosphatase inhibitors. Protein extracts were analyzed by immunoblotting as described above. Antibodies used for immunoblotting assays are listed in **Supplementary Table 3**.

### Quantitative Reverse Transcription PCR (RT-qPCR)

RNA was extracted using RNAeasy mini kit (Qiagen) according to the manufacturer’s instructions. Reverse-transcription was performed with High-capacity cDNA reverse transcription kit (Applied Biosystems), and cDNA was analyzed by quantitative real-time PCR (qPCR) on an ABI-PRISM 7500 Fast Real-Time PCR system (Applied Biosystems). PCR reactions were performed in 25 μL of diluted cDNA (10X dilution), 0.3 μmol of each primer and 12.5 μL of TB Green Premix Ex Taq (Takara). Oligonucleotides used for RT-qPCR are listed in **Supplementary Table 4**. All RT-qPCR were performed in duplicate. To allow comparison between samples, transcript quantification was performed after normalization with ABL gene using the ΔCt method and calculated according to the following formula 2^(CtABL-Ctgene)^.

### Statistical Analysis

Statistical analyses were performed using GraphPad Prism software (GraphPad Software). Significance was evaluated by 2-tailed Mann-Whitney *U* test. A *P* value inferior to 0.05 was considered significant.

## Results

### scRNA-Seq Analysis Identifies Lower Calcium Pathway Activity in Pten^del^-Specific DP Clusters

**Figure 1:**
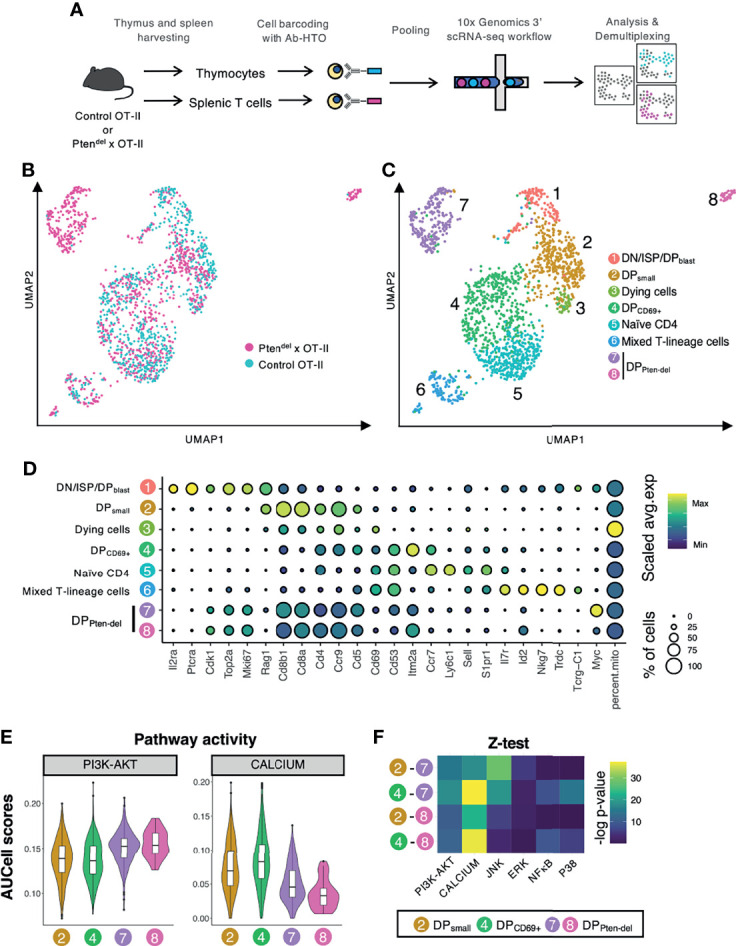
Single-cell RNAseq analysis reveals an impact of PTEN loss on calcium pathway. **(A)** Overview of scRNAseq workflow. The scRNAseq assay was performed with thymus and spleens from 4 weeks-old Control OT-II and Pten^del^×OT-II mice. Thymocytes and splenic T cells were harvested, each sample was labelled with a distinct Ab-HTO, pooled and then processed for scRNAseq using the 10X Genomics approach. Following sequencing, reads were assembled, quantified, normalized and demultiplexed to identify the original sites and samples. **(B, C)** UMAP plots of cleaned scRNAseq dataset of thymus and splenic T cells (1686 cells in total) from Control OT-II and Pten^del^×OT-II mice. The UMAP plot is colored according to mouse genotype **(B)** or to the 8 clusters **(C)**. **(D)** Dot plots showing the expression level of established marker genes of T cell differentiation. Dot color represents the scaled average expression of the specified gene across the various clusters, and dot size indicates the percentage of cells expressing the specified gene. **(E, F)** Analysis of pathways activity. Genes lists (**Supplementary Table 2**) corresponding to pathways of interest were retrieved from MSigDB database and were scored in DP clusters using AUCell. **(E)** Violin plots reporting AUCell score for PI3K/AKT and calcium pathways activity in clusters 2, 4, 7 and 8. **(F)** Heatmap of –log p-value calculated by the z-test of pathway activity, for two by two combinations of the four clusters indicated on the left. The analyzed pathways are indicated at the bottom of each column.

We applied a single-cell RNA sequencing approach (scRNAseq) to analyze the dynamical process of physiological/pathological thymocyte differentiation and to investigate the role of PTEN in this process. Herein we used Pten^del^×OT-II mice corresponding to Pten^del^ mice crossed with the OT-II transgenic mouse model. In the latter, OT-II corresponds to a fit transgenic Vα2/Vβ5.1 TCR recognizing the chicken Ovalbumin antigen in the context of MHC-II molecules (14). Similarly to Pten^del^ mice, Pten^del^×OT-II mice develop TCRαβ^+^ T-ALL at approximately 11 weeks of age (11). ScRNAseq assays were performed with one Pten^del^×OT-II mouse and one Control OT-II mice of 4 weeks of age. The PTEN-deficient mouse did not display clinical signs of disease at the time of dissection: spleen was of normal size, yet thymus was slightly enlarged. Accordingly, FACS analysis showed that conversely to physiological control thymocytes, a large part of DP (CD4^+^CD8^+^) cell population is TCR positive in Pten^del^×OT-II mouse (**Supplementary Figure 1A**) suggesting a pathological onset. This phenotype (high proportion of DP TCR^+^ cells) is recurrently observed in pre-leukemic Pten^del^ mice both in OT-II and non OT-II backgrounds (**Supplementary Figures 1B, C**). Thymocytes and splenic T lymphocytes samples were each marked with a distinct anti-MHC class I/CD45-hashtag oligonucleotide (HTO) antibodies, then pooled and finally analyzed by scRNAseq using 3’ 10X Genomics technology (**Figure 1A**). After sequencing data pre-processing, which includes the removal of poor quality cells, of cell-doublets and non-T cells (see *Materials and Methods*, *Single-Cell RNA Sequencing Data Analysis*), we projected the data using the UMAP non-linear dimensionality reduction method that enabled us to visualize cell transcriptome heterogeneity (17) (**Figure 1B**). We identified 8 clusters and assigned them to cell type according to established markers of thymopoiesis (19–23) (**Figures 1C, D**; see also **Supplementary Figure 2** for unsupervised cluster gene markers). Cluster 1 encompasses DN, ISP and DP_blast_ cells, cluster 2 corresponds to DP_small_, cluster 4 to DP_CD69+_ cells, and cluster 5 to naive CD4^+^ cells. Cluster 3 comprises dying cells that are featured by high proportion of mitochondrial transcripts. Cluster 6 named ‘mixed T-lineage cells’ includes activated CD4 SP, CD8 SP and γδ T cells. Splenic CD4 and CD8 T cells are found in clusters 5 and 6 (**Supplementary Figure 3**). Finally, two clusters (7 and 8) comprising mainly Pten^del^ DP cells were detected. These two Pten^del^-specific DP clusters are labelled DP_Pten-del_ clusters.

We assessed *Myc* expression in our scRNA-seq dataset. Indeed, in human T-ALL, PTEN loss correlates with the accumulation of MYC protein in leukemic blasts (29). The functional interaction between MYC and PTEN is also sustained by mouse models showing that inactivation of PTEN in thymocytes leads to leukemic cells over-expressing MYC (6, 8, 11). Therefore, high level of Myc expression is a reliable marker of PTEN loss-mediated leukemogenesis. *Myc* mRNA is detected in DN/ISP/DP_blast_ cluster (cluster 1), which was expected as *Myc* is highly expressed in physiological DN thymocytes (21). *Myc* is also strongly expressed in cluster 7 and in lesser extent, in the small cell cluster 8 (**Supplementary Figure 4**). This indicates leukemia onset in these two Pten^del^-specific DP clusters.

Next, in order to better characterize the two DP_Pten-del_ clusters we inferred pathway activities. To do so, we retrieved gene sets of pathways of interest from Molecular Signature Database (mSigDB) (**Supplementary Table 2**) and scored their activities in our scRNA-seq dataset using AUCell (25). We compared inferred activity levels of clusters 2 and 4 (physiological cells) and clusters 7 and 8 (Pten^del^-specific DP cells). Consistent with the established PTEN’s function as the main PI3K antagonist (30) PI3K/AKT pathway is significantly up-regulated in DP_Pten-del_ clusters (**Figure 1E**). Among the five others TCR downstream pathways analyzed: Calcium, ERK, P38, JNK and NFκB (**Figure 1E** and **Supplementary Figure 5**), the calcium pathway displays the most significant p-value from statistical analysis by z-test (**Figure 1F**).

### TCR-Induced Calcium Signaling Is Abrogated in PTEN Deficient T-ALL

**Figure 2:**
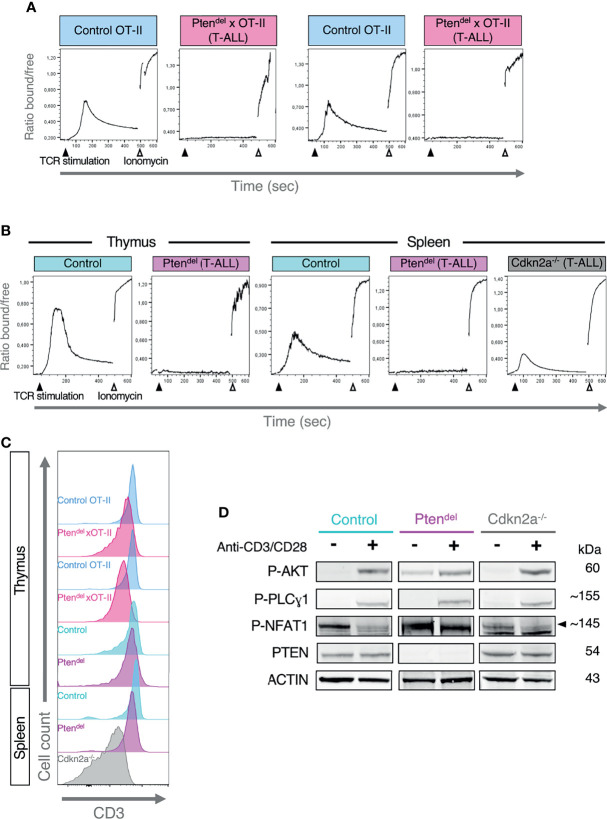
Inhibition of calcium signaling in Pten^del^ T-ALL. **(A, B)** Flow cytometry analysis of Calcium flux. Thymocytes from Control mice or T-ALL cells previously loaded with Indo-1 AM and stained with anti-CD4 and anti-CD8 antibodies, were incubated one minute with biotinylated anti-CD3 antibody and then analyzed by flow cytometry for 10 minutes. Fifty seconds following the start of acquisition, TCR stimulation was induced by addition of streptavidin, and at 8 min, ionomycin (a calcium ionophore) was added. The plots display the evolution over time of the ratio between two wavelengths, 410 nm and 475 nm corresponding to Indo-1 bound to Ca^2+^ and free Indo-1, respectively. The mouse model used is indicated on the top of each plot. **(A)** The assays were performed with OT-II mouse models in duplicate. CD4 SP thymocytes from Control OT-II or leukemic Pten^del^ × OT-II mice (aged between 10 and 12 weeks) were analyzed. **(B)** The assays were carried out with Control, Pten^del^ and *Cdkn2a^-/-^* mouse models. Plots shown are representative of 3 independent experiments. For Control and Pten^del^ mice, thymic and splenic CD4 SP cells were analyzed. For *Cdkn2a^-/-^* model, splenic T-ALL cells were analyzed. **(C)** Flow cytometry histograms showing CD3 expression in cells analyzed in panels **(A, B, D)** Analysis of early TCR signaling by immunoblotting. A representative case of Pten^del^ T-ALL (n=3), *Cdkn2a^-/-^* T-ALL (n=3) and control thymocytes (n=3) are shown. Cells from total thymus (Pten^del^ and control mice) or from total spleen of NSG mice (*Cdkn2a^-/-^* mouse model) were unstimulated (-) or stimulated (+) with anti-CD3/CD28 antibodies for 2 minutes and analyzed by immunoblotting with antibodies specific for phosphorylated AKT S473 (P-AKT), phosphorylated PLCγ1 at Y783 (P-PLCγ1), phosphorylated NFAT1 at S54 (P-NFAT1), PTEN and ACTIN. **(B, D)** Control and Pten^del^ mice were aged between 10 to 15 weeks.

Calcium signaling pathway is swiftly induced following TCR engagement. Indeed, upon TCR stimulation, LCK phosphorylates CD3 molecules, providing a docking site for ZAP70 which is then phosphorylated by LCK. In turn, ZAP70 phosphorylates and activates LAT, this generates a multimolecular platform (LAT signalosome) comprising several adaptors and effectors molecules, which insures the propagation of TCR signals. Among LAT-binding proteins, the phospholipase Cγ1 (PLCγ1) catalyzes the production of diacylglycerol (DAG) and inositol (1, 4, 5)-trisphosphate (IP3). IP3 binds to IP3 receptors (ITPR also known as IP3R), which are IP3-gated Ca^2+^ channel localized on the endoplasmic reticulum (ER), this induces calcium release from ER into the cytosol and triggers the activation of several downstream signaling effectors (31). Thus, to assess the potential impact of PTEN loss on the calcium signaling pathway, we performed *ex vivo* TCR stimulation assays and monitored calcium flux by flow cytometry (**Figures 2A, B**). We used Indo-1 AM compound that is a ratiometric and sensitive indicator dye for measuring intracellular calcium. Upon Indo-1 AM entry in the cell, intracellular esterase cleaves the acetyloxymethyl (AM) groups rendering Indo-1 membrane-impermeant. Then, following UV-excitation, Indo-1 fluoresces at different wavelengths depending on whether it is bound to calcium (~410 nm) or free (~475 nm), thus the ratio of these two wavelengths allows to investigate modifications in intracellular calcium concentration. As expected, TCR stimulation with anti-CD3 antibodies of CD4 SP thymocytes from Control or Control-OT-II mice leads to a strong increase of cytosolic Ca^2+^ (**Figures 2A, B**). In a sharp contrast, TCR stimulations of PTEN-deficient leukemic blasts do not induce Ca^2+^ release and the same result is observed for both OT-II and non OT-II mouse models (**Figures 2A, B**). Furthermore, we showed that CD3 is expressed at similar levels at the cell surface of control CD4 SP thymocytes and Pten^del^ T-ALL (**Figure 2C**). These results indicate that Ca^2+^ flux inhibition is not due to a specific effect of OT-II transgenic system nor to an absence of the TCR complex.

We then monitored calcium flux using a *Cdkn2a^-/-^* mouse model of T-ALL that has been previously shown to be PTEN-proficient (11). Compared to Pten^del^ T-ALL, CD3 expression is lower in *Cdkn2a^-/-^* leukemic blasts (**Figure 2C**) nevertheless anti-CD3 stimulation of *Cdkn2a^-/-^* leukemic cells induces a release of Ca^2+^ (**Figure 2B**). In the same line, TCR stimulation of cells from pre-leukemic Pten^del^ mice induces a normal calcium flux (**Supplementary Figure 6**), suggesting that the oncogenic landscape shaped by PTEN loss is necessary to impair calcium signaling. The absence of a defective calcium signaling pathway in non-tumor Pten^del^ thymocytes is also supported by the analysis of pathways activities in our scRNA-seq dataset. Indeed, non-tumor Pten^del^×OT-II cells displays similar levels of calcium signaling activities compared to Control OT-II cells (**Supplementary Figure 7**).

Next, we undertook to define whether the absence of calcium flux results from a defect in proximal TCR signaling. Thus, control thymocytes and leukemic cells were freshly harvested from mice and were stimulated for 2 minutes using biotinylated anti-CD3 and anti-CD28 antibodies. Then, protein extract from unstimulated and stimulated cells were analyzed by immunoblotting with specific antibodies (**Figure 2D**). First, we assessed the activation of AKT pathway by monitoring AKT phosphorylation at Ser473. AKT is the main downstream target of PTEN, accordingly we observed in unstimulated Pten^del^ T-ALL cells, a basal level of P-AKT that is higher than in control cells. Yet, and as previously shown (11), upon TCR stimulation, the increase of P-AKT species is similar in Pten^del^ T-ALL than in Control cells. Then we investigated the activation of PLCγ1, which is in charge to produce IP3, thus we monitored phosphorylation of PLCγ1 at Tyr783 (P- PLCγ1). In control cells, TCR stimulation using anti-CD3/CD28 antibodies induces a marked increase of P-PLCγ1 species. Yet, a similar induction was observed with Pten^del^ T-ALL indicating that TCR proximal signaling leading to PLCγ1 phophorylation and thus to the production of DAG and IP3, is not affected in those leukemic cells. NFAT factors are the main downstream effectors of calcium signaling. Indeed, the cytosolic calcium influx induces the dephosphorylation of NFAT proteins, permitting NFAT to translocate into the nuclei and to fulfill its function as a transcription factor. We assessed phosphorylation status of NFAT1 and found that CD3/CD28 stimulation induces a marked decrease in the level of NFAT1 P-Ser54 species in control cells, while this level remains unchanged in Pten^del^ T-ALL samples.

Altogether, our data show that in PTEN-deficient T-ALL, TCR-induced Ca^2+^ flux is fully abolished and consequently, the downstream effector of calcium signaling, NFAT, is impaired.

### Logical Modelling of Calcium Flux in Thymocytes

**Figure 3:**
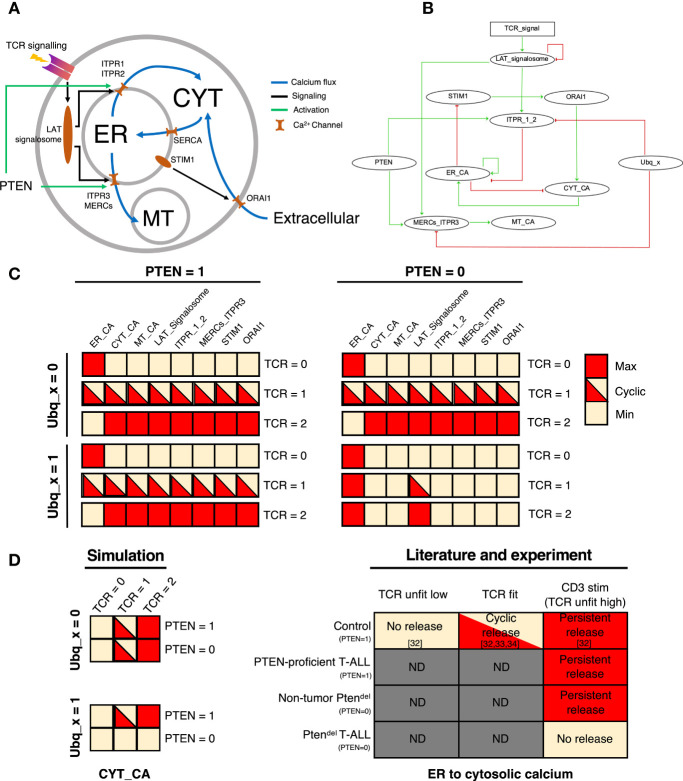
Mathematical modelling of calcium flux and analysis of the PTEN impact. **(A)** Schematic representation of TCR-induced calcium flux in thymocytes. The flow of calcium between the three major calcium compartments, endoplasmic reticulum (ER), cytosol (CYT), and mitochondria (MT) is controlled by calcium channels, such as ITPR1, ITPR2, SERCA, ORAI1, ITPR3 and MERCs with the flow direction indicated by blue arrows. Upon stimulation, TCR signaling is amplified by the LAT signalosome that notably yields to the production of IP3 that binds to its receptors (ITPR) leading to the release of Ca^2+^ from ER to cytosol. The drop of Ca^2+^ in the ER induces a conformational change of STIM1 that promotes the binding of STIM1 to ORAI1, yielding to calcium fluxes from extracellular matrix to cytoplasm. Following Ca^2+^ release from ER to cytosol, the active transport system mediated by sarcoplasmic/ER Ca^2+^-ATPases (SERCA) can sequester calcium back into ER. The putative interactions of PTEN with ITPR are indicated by green. **(B)** Logical regulatory network representing the thymocyte specific TCR-activated calcium signaling. Nodes of the network represent calcium signaling components (ellipse nodes for Boolean node, rectangular for multilevel). Green edges stand for the activations, red ones for the inhibitions (logical functions are given in **Supplementary Table 5**). **(C)** Attractors of the logical model for each combinations of inputs: simulations with PTEN=1 and PTEN = 0 are represented in the left and right columns respectively, Ubq_x =0 (top) and Ubq_x = 1(bottom), and in each situation are displayed the 3 values of the TCR (0/1/2). Colors represent the activity levels, red for active (1), yellow inactive (0). Bicolored cases represent an oscillating node (cyclical attractor). **(D)** Comparison of cytosol state simulated with the model, and biological data extracted from literature [control, see references (32–34)] and experiments (Pten^del^: non-tumor and T-ALL; PTEN-proficient T-ALL that corresponds to *Cdkn2a^-/-^* T-ALL) according to CD3 stimulation (see **Figure 2** and **Supplementary Figure 6**). ND means not determined.

To understand the role of PTEN in the TCR-induced calcium signaling, we constructed a logical model providing a schematic view of the regulation of calcium flux between the different intracellular compartments (**Figure 3A**). The model encompasses the three major calcium reservoirs, represented by three nodes: ER_CA (endoplasmic reticulum), CYT_CA (cytosol), and MT_CA (mitochondria). Fluxes of calcium between these three components are controlled by calcium channels and regulators including, STIM1, ORIA1, ITPR_1_2 (ITPR subtype 1 and 2), MERCs_ITPR3 (ITPR3 within mitochondrial ER contact sites or MERCs) (**Figure 3B**). The TCR activation is represented by an input node of the model, and its associated variable can take three values standing for unfit low TCR activation (0), a fit TCR activation (1), and an unfit high TCR activation (2). These three values are related to the differential TCR signal strength that gives rise to the three types of thymocyte outcomes during positive and negative selection, namely death-by-neglect for unfit low TCR, survival and differentiation for fit TCR and negative selection for unfit high TCR. We also integrated PTEN as an input node. Its role on calcium signaling has been very little reported in the literature. PTEN has been described to prevent the degradation of ITPR3, therefore the loss of PTEN eventually leads to the loss of ITPR3 (35). Hence, we introduced PTEN as an activator of ITPR3. Moreover, the binding region of PTEN in ITPR3 is well conserved in ITPR1 and ITPR2 (**Supplementary Figure 8**), with high protein sequence similarity of over 85% in both, that may suggest an activation of PTEN on ITPR_1_2. We performed simulations of two versions of the model, with and without the activation of PTEN on ITPR_1_2. The model with both activations provides more realistic results so we decided to keep the activation of IP3R_1_2 by PTEN (**Supplementary Figure 9**). Finally, we introduced the input node Ubq_x which refers to the presence of an ubiquitin ligase that inhibits ITPR proteins. Indeed, Kuchay et al. also described that, in fibroblasts, PTEN competes with the ubiquitin ligase FBXL2, for the regulation of ITPR3 protein level (35). However, the ubiquitin ligase involved in ITPR degradation in thymocyte, remains to be characterized. Logical equations depicting the local dynamics are given in **Supplementary Table 5**.

Simulations of the model provide the different scenarios for each combination of input values (Ubq_x, PTEN and the TCR activation). Attractors of the dynamics stand for the asymptotical behaviors of the system, i.e. a set of states in which the system is stabilized. For each of the 6 combinations of input values, the system presents a unique attractor. When PTEN = 0 and Ubq_x = 1, the system reaches a stable state with ER_CA = 1 and all other components inactive, only the LAT_signalosome node varies according to the TCR signal (**Figure 3C**). In the others three combinations of PTEN and Ubq_x values, the system is stabilized in a state depending on the TCR activation (**Figure 3C**). When TCR=0 (no activation), the system reaches a stable state with all components inactive except ER_CA. When TCR=1, a cyclical attractor with all components cycling, except ER_CA which is inactive. Finally, the simulation with TCR=2 leads to a stable state with all components active, except ER_CA which is inactive. When PTEN is absent (PTEN=0), whatever the TCR signal, the system reaches the stable state with ER_CA=1 and all other components inactive (**Figure 3C**).

When PTEN is present, which is the case for Control thymocytes or PTEN-proficient T-ALL, we observed a calcium release (**Figures 2A, B**) which is consistent with the results of PTEN = 1 simulation (whatever the state of Ubq_x). Also, those results are in line with the calcium dynamics described by Melichar et al. study (32), which depicted an oscillating cytosolic calcium flux for thymocytes with a fit TCR (positively selected) and a prolonged calcium flux for thymocytes harboring unfit high TCR (negatively selected) (**Figure 3D**). Hence, TCR activation triggers the calcium fluxes and when there is no TCR activation, majority of cellular calcium is stored in the ER. When PTEN = 0, we have two behaviors depending on the state of Ubq_x. The results of simulations with Ubq_x = 1 and PTEN = 0 are in line with our data obtained with Pten^del^ T-ALL samples, showing the complete blockage of calcium flux upon TCR stimulation (**Figure 2**). Moreover, we showed that in Pten^del^ thymocytes, calcium from the ER can be released upon ionomycin treatment, indicating the presence of Ca^2+^ in the ER, which is also captured by the model simulation. In the other hand, calcium flux is not impaired in non-tumor PTEN deficient thymocytes (**Supplementary Figure 6**) and this is consistent with the simulation with Ubq_x = 0 and PTEN = 0. Altogether these data suggest that activation of the ubiquitin ligase may be dependent on the oncogenic landscape.

### Levels of ITPR Proteins Are Reduced in PTEN-Deficient T-ALL

**Figure 4:**
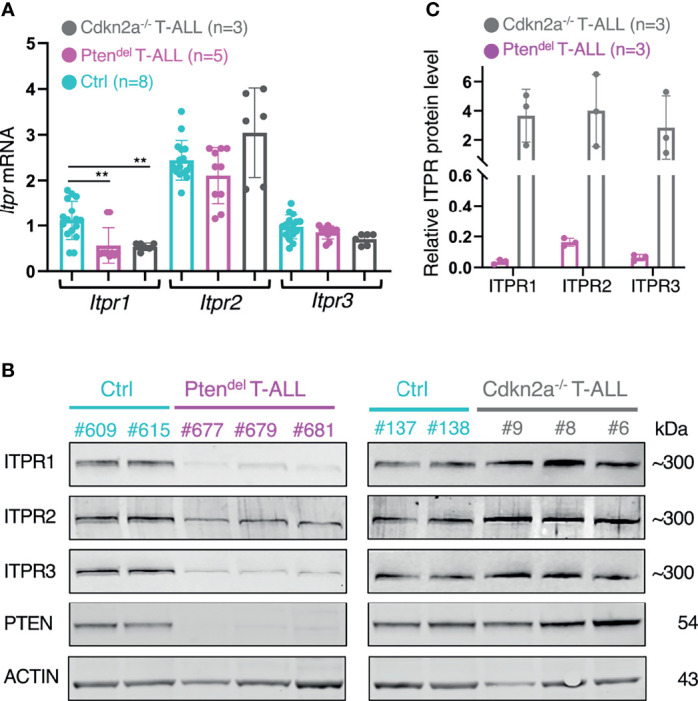
PTEN-deficiency correlates with a down regulation of IP3 receptors expression. **(A)** Quantification of *Itpr1, Itpr2* and *Itpr3* mRNA expression by RT-qPCR performed on cDNA obtained from total thymus of Control (Ctrl) mice (n = 8) and of leukemic Pten^del^ mice (n = 5), and total spleen from leukemic NSG mice engrafted with distinct *Cdkn2a^-/-^* T-ALL (n = 3). Control and Pten^del^ mice were aged between 10 and 15 weeks. The assay was performed in duplicate. Transcripts levels were normalized to ABL. Error bars show means with SD. Statistical significant difference was assessed using Mann-Whitney test (**P< 0.01). **(B)** Analysis of ITPR proteins expression. Immunoblotting assays were performed with antibodies specific for ITPR1, ITPR2, ITPR3, PTEN and ACTIN as a loading control. Total thymic cells from disease-free Control (Ctrl) and leukemic Pten^del^ mice were analyzed. Leukemic cells from *Cdkn2a^-/-^* model were harvested from spleen of NSG mice. The identification of analyzed mice or *Cdkn2a^-/^* ^-^ T-ALL is indicated (#number). **(C)** Quantification of ITPRs protein expression levels. The bands of interest in immunoblots shown in panel **(B)** were quantified and values of ITPR bands were first normalized to ACTIN. Then ITPR/ACTIN values of the Pten^del^ and *Cdkn2a^-/-^* samples were normalized to the mean values of Control samples.

In order to validate our mathematical model suggesting that PTEN may interact functionally with ITPR proteins, we undertook to assess mRNA and protein expression of IP3 receptors. Thus, using RT-qPCR and immunoblotting we analyzed control thymocytes and leukemic cells from Pten^del^ and *Cdkn2a^-/-^* T-ALL. We found that *Itpr2* and *Itpr3* mRNA are expressed at similar levels in all samples, while *Itpr1* is slightly more expressed in Control cells than in leukemic cells. However, *Itpr1* expression is alike in both Pten^del^ and *Cdkn2a^-/-^* T-ALL (**Figure 4A**). In contrast to mRNA, quantities of ITPR1 and ITPR3 proteins are drastically reduced in Pten^del^ T-ALL compared to Control thymocytes or *Cdkn2a^-/-^* T-ALL (**Figures 4B, C**). In a lesser extent, ITPR2 protein level is also decreased in Pten^del^ T-ALL. In line with calcium flux assays (**Supplementary Figure 6**), ITPRs protein levels are not markedly decreased in pre-leukemic Pten^del^ thymocytes (**Supplementary Figure 10**).

Collectively, our data indicate that quantities of IP3 receptors are strongly reduced in PTEN deficient T-ALL, and we conclude that such inhibition of ITPR proteins expression is likely at the basis of the calcium flux abrogation in these Pten^del^ leukemic cells.

## Discussion

PTEN-deficient T-ALL account for 15-20% of T-ALL cases and are usually associated with mature TCRαβ^+^ subgroup (2, 36). Using mouse models, we previously showed that in a PTEN-deficient context, T-ALL onset is dependent on the nature of the TCRαβ (11). Herein, we undertook to better understand the functional interaction between PTEN and TCR signaling in leukemia. First, the analysis of scRNAseq data obtained from Pten^del^ thymocytes undergoing leukemogenesis and from PTEN-proficient control thymocytes, suggests that Ca^2+^ signaling pathway might be impacted in Pten^del^ T-ALL (**Figure 1**). We validated this observation by carrying out calcium flux assays. Indeed, we showed that upon TCR stimulation, PTEN-deficient T-ALL do not release Ca^2+^ into the cytosol (**Figure 2**). This full abrogation of calcium flux is as severe as the one described when a major component of TCR signaling is inactivated, such as LAT (37) and ZAP (38). Therefore, our result is consistent with our previous study (11) suggesting that TCR signaling is impaired in PTEN deficient T-ALL.

TCR signaling involves multiple components that trigger the activation of several downstream signaling effectors (31). In this context ITPR channels are crucial factors. Upon binding IP3 molecules, they release Ca^2+^ that activates Calcineurin/NFAT pathway, which is then endowed to turn on its transcriptional program (39).

Among the ITPR protein family, ITPR3 is described as a Ca^2+^ channel between ER and mitochondria and is part of MERCs (40). Yet the mathematical simulation of our model harboring this positive interaction between PTEN and ITPR3-MERC did not recapitulate the abrogation of calcium flux. Thus, we hypothesized that PTEN interacts with others ITPR proteins and integrated in our model a putative interaction between PTEN and ITPR1/2. Using immunoblotting we uncovered that *Pten* deletion in T-ALL cells correlates with a decrease of the three types of ITPR proteins. This result is different from Kuchay et al. study indicating that only ITPR3 is impacted by PTEN loss (29). However, this study analyzed primary fibroblasts, HeLa and COS-7, and in the proposed scenario, PTEN impedes ITPR3 degradation by competing with the ubiquitin ligase FBXL2. The authors did not find any FBXL2 binding region in ITPR1 and ITPR2, yet according to protein sequence analysis, the putative PTEN binding region described for ITPR3 is partially conserved (above 85% similarity) in ITPR1 and ITPR2. Therefore, we speculate that, in thymocytes, another ubiquitin ligase may replace FBXL2 to compete with PTEN for ITPR1/2 degradation. We also observed that calcium flux and expression of ITPR proteins are not impaired in non-tumor Pten^del^ T cells, indicating a role of the oncogenic landscape. As reported by Kuchay et al. (35), regulation of ITPR protein level may rely on the competition between PTEN and an ubiquitin ligase. Therefore, our hypothetical scenario, which is also conveyed by our mathematical model, is that, such ubiquitin ligase would be induced in T-ALL samples, favoring ITPR proteins degradation especially in the context of PTEN loss. However, the full molecular mechanism of the regulation of ITPR protein level in thymocytes, and notably the ubiquitin ligase involved remains to be characterized.

It has been previously shown that inactivation of all three ITPR subtypes in mouse thymocytes gives rise to T-ALL (41). In Ouyang et al. study, *Itpr* genes encoding for the 3 ITPR subtypes were simultaneously deleted at the DN1 stage of thymopoeisis, this led to a defect in the developmental progression of thymocytes toward DP stage and the sustain expression of NOTCH transcriptional targets which is likely at the basis of T-ALL onset (41). ITPR proteins together with Ca^2+^ channels/pumps, and exchangers are part of the Ca^2+^ signaling toolkit (42) which insures the regulation of Ca^2+^ homeostasis and orchestrates specific cellular processes. Alteration in the expression of the toolkit components can affect cell fate and be linked to oncogenic pathway (43). Our study suggests that in the context of PTEN loss-mediated leukemogenesis down-regulation of IP3 receptors expression is part of the oncogenic landscape. Yet, the contribution of the abrogation of TCR-induced calcium flux in leukemic development of thymocytes awaits future investigations.

Here we constructed a mathematical model to uncover the functional links between TCR, PTEN and calcium flux. Several logical models have depicted TCR signaling (44–47). Yet, in contrast to all these previous models designed for peripheral T cells, our model is adapted to thymocytes, as it takes into account the strength of TCR signaling, which is a crucial feature for thymic selection. Our model captures the calcium flux dynamics described in the literatures (32–34), and highlights the impact of PTEN. Our analysis suggests that PTEN may play a crucial role in the TCR signaling puzzle, in which calcium subnetwork represents an important piece. Moreover, the calcium signaling interacts with other pathways such as ERK signaling (48) and reactive oxygen species (49). Thus, building-up an extended mathematical model comprising such pathways and modelling their crosstalk may contribute to decipher the role of PTEN in physiological and pathological development of thymocytes.

## Data Availability Statement

The data presented in the study are deposited in the NCBI GEO and NCBI SRA repositories, accession numbers GSE186498 and SRP342978 respectively.

## Ethics Statement

The animal study was reviewed and approved by French ethical committee (project APAFIS#4484-2016031113534101).

## Author Contributions

CG, MN, JQ, ML, and DP-B performed the biological experiments and analyzed the data. SP and DP performed bioinformatics analysis. SP and ÉR constructed and analyzed the mathematical model. SP, DP, ÉR, and DP-B wrote the paper. All authors read and approved the final manuscript.

## Funding

This study was partly supported by research funding from the Canceropôle PACA, Institut National Du Cancer and Région Sud. SP received funding from the European Union’s Horizon 2020 research and innovation program under the Marie Skłodowska-Curie Grant Agreement No. 713750, and from the Regional Council of Provence-Alpes-Côte d’Azur, A*MIDEX (No. ANR11-IDEX0001-02). This project received financial support from ITMO Cancer of AVIESAN (Alliance Nationale pour les Sciences de la Vie et de la Santé, National Alliance for Life Sciences & Health) within the framework of the Cancer Plan (project n°C19046S) and from CNRS ‘Osez l’interdisciplinarité!’ program-’DMATh’ project.

## Conflict of Interest

The authors declare that the research was conducted in the absence of any commercial or financial relationships that could be construed as a potential conflict of interest.

## Publisher’s Note

All claims expressed in this article are solely those of the authors and do not necessarily represent those of their affiliated organizations, or those of the publisher, the editors and the reviewers. Any product that may be evaluated in this article, or claim that may be made by its manufacturer, is not guaranteed or endorsed by the publisher.
