# Supplementary material for: Calcium Signaling Is Impaired in PTEN-Deficient T Cell Acute Lymphoblastic Leukemia
Source: Front Immunol. 2022 Feb 2;13:797244. doi: 10.3389/fimmu.2022.797244 (PMC8847596; doi:10.3389/fimmu.2022.797244)
Supplement: Supplementary file 1 [file DataSheet_1.pdf]

Supplementary Material

Supplementary Figures

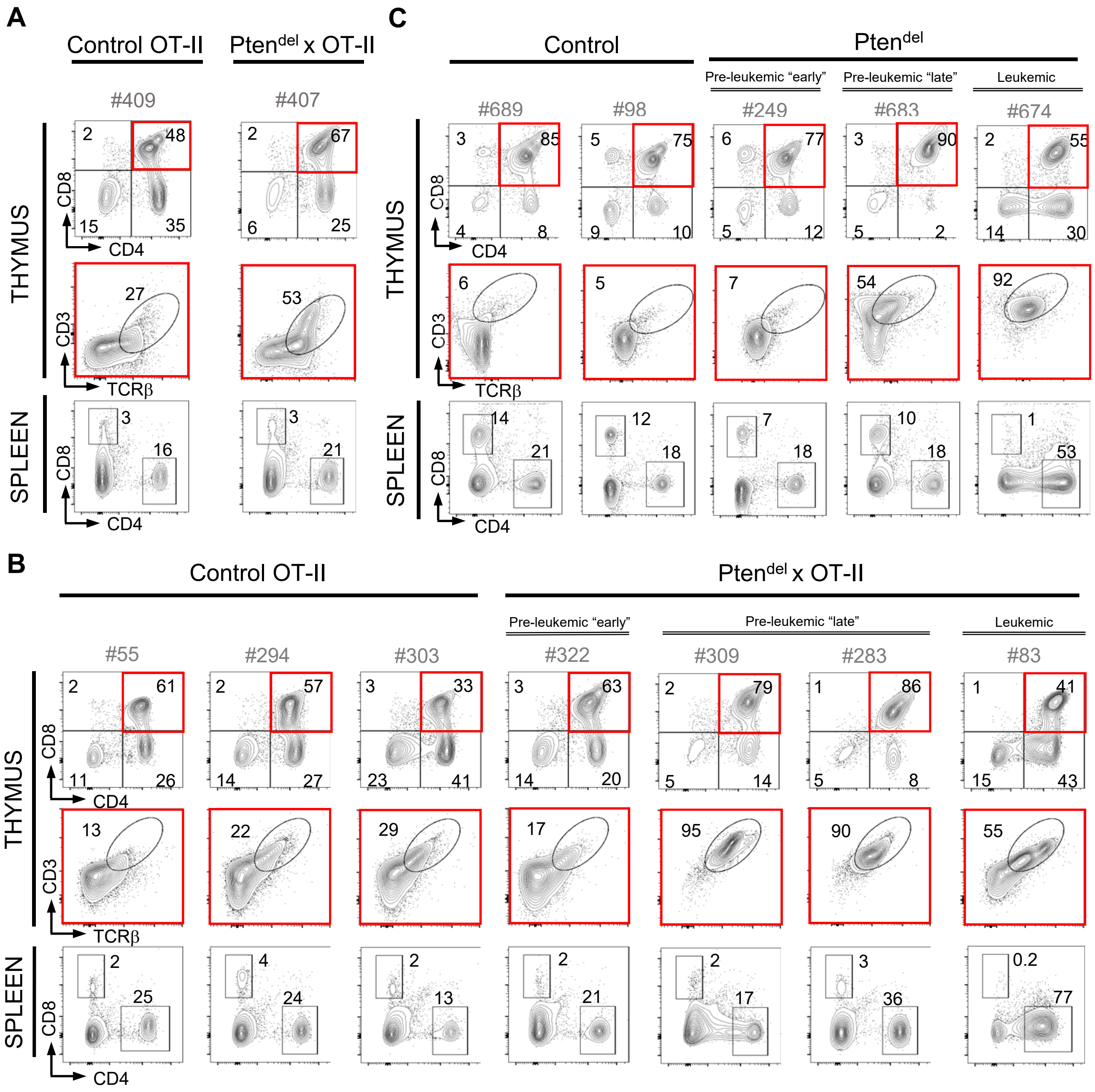

Supplementary Figure 1. Phenotypes of thymus and spleens of Pten^{del} mice from different genetic backgrounds

(**A, B, C**) FACS contour plots showing CD4 and CD8 expression on thymocytes (top panels) and splenocytes (bottom panels) from the indicated mice. Percentages of cells in depicted gates are shown. In the middle panels, the FACS contour plots show CD3 and TCRβ expression on DP thymocytes. The percentage of TCR positive cells is indicated. The identification (#number) of analyzed mice is shown. **(A)** Phenotypes of the control OT-II and Pten^{del} x OT-II mice used for scRNA-seq analysis. **(B-C)** Phenotypes of additional control and Pten^{del} mice in OT-II background **(B)** and non OT-II background mice **(C)**. Analysis were performed at the different stages from disease-free to full-blown leukemia. In pre-leukemic stages, mice do not display clinical signs of leukemia and the spleen is not invaded by leukemic blasts. Typically in the 'pre-leukemic early' stage, thymus from Pten^{del} mice are similar to Control thymus. While in 'pre-leukemic late' stage a large part of DP cells are TCRαβ^{+} and the thymus contains tumor blasts.

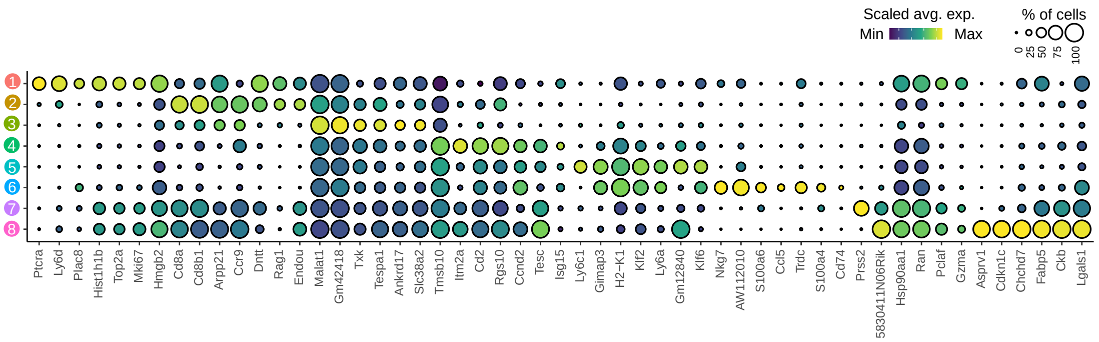

Supplementary Figure 2. Expression of marker genes across clusters

Dot plot showing the expression level of top 7 marker genes of each cluster. Dot size represents the percentage of cell expressing the gene of interest, while dot color represents the average expression of the gene of interest across the 8 clusters.

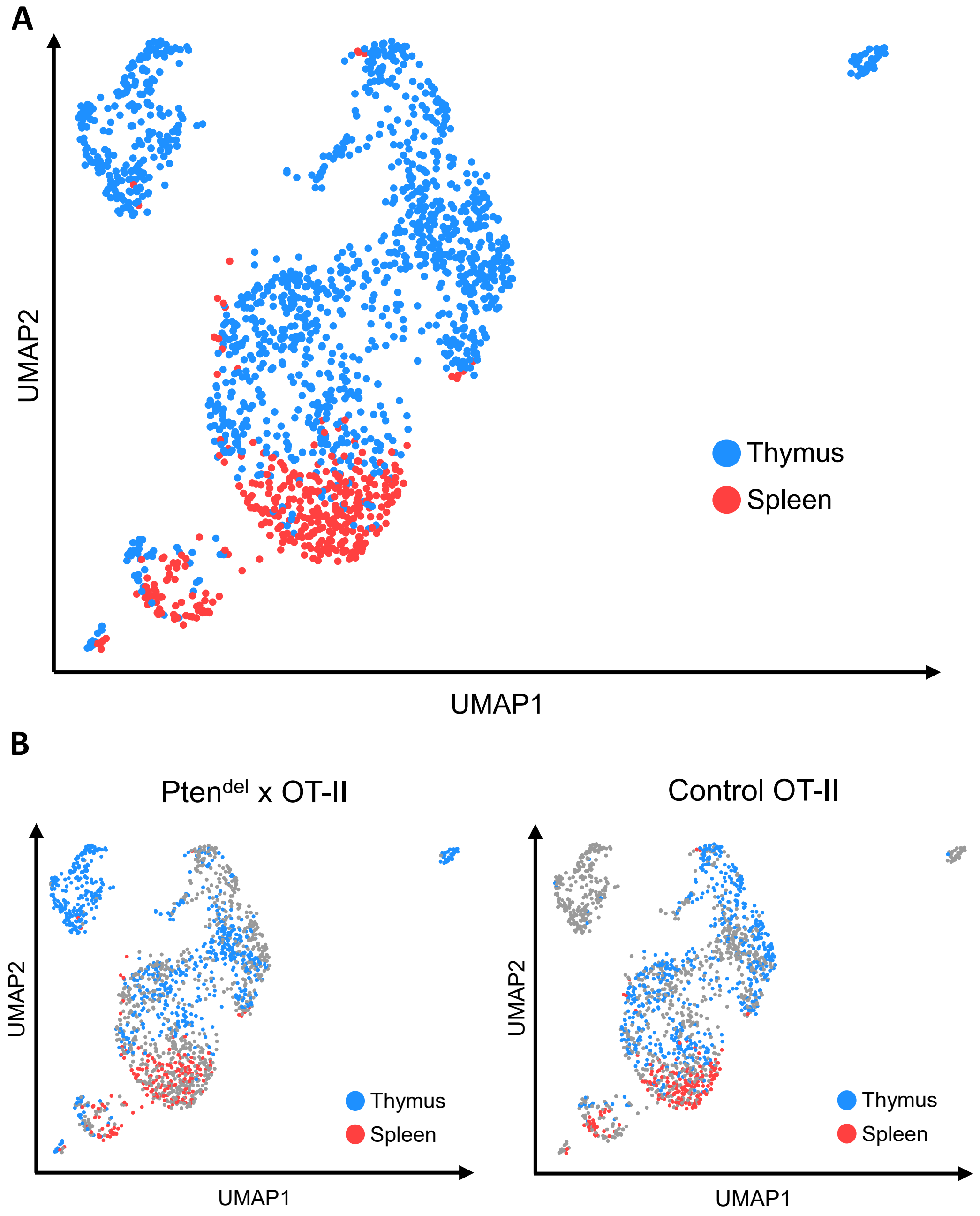

Supplementary Figure 3. Visualization of thymic and splenic cells

(A) UMAP plot colored according to tissue of origin. **(B)** Same as panel A except that each mouse genotype is highlighted.

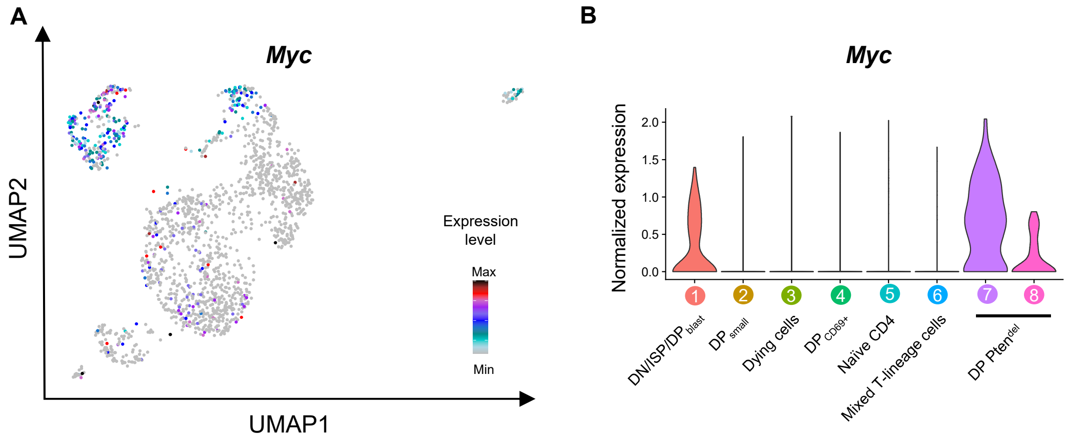

Supplementary Figure 4. Cells from Pten^{del}-specific DP clusters express *Myc*

(A) UMAP plot colored according to the scaled *Myc* mRNA expression. **(B)** Violin plots of normalized *Myc* expression for the 8 annotated clusters.

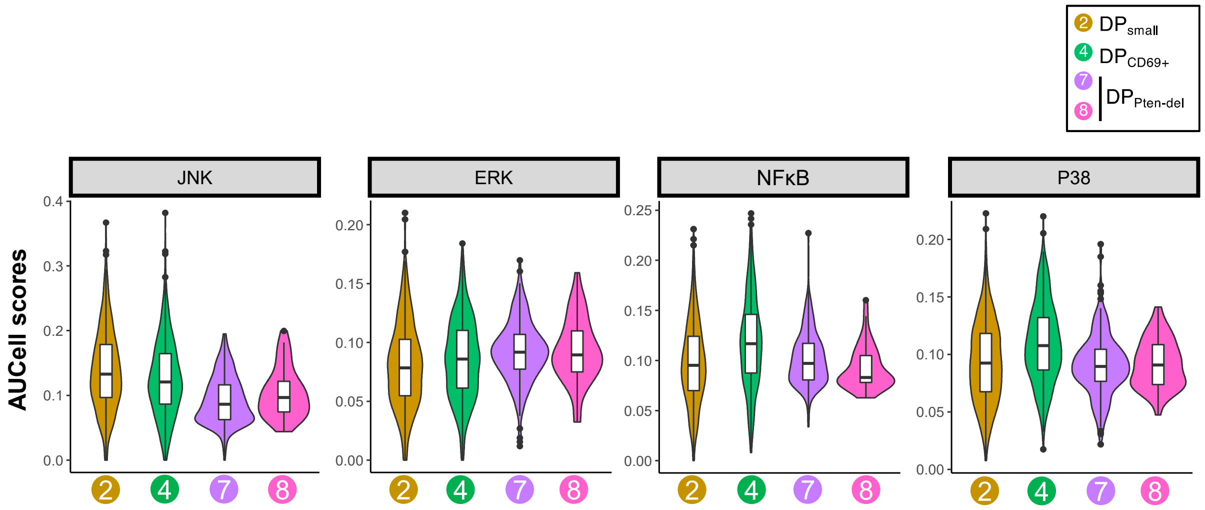

Supplementary Figure 5. Inferred JNK, ERK, NFκB and P38 pathways' activities using scRNAseq data

Violin plots displaying AUCell scores for the inferred activity of selected pathways (ERK, P38, JNK, and NFκB) in DP clusters (clusters 2, 4, 7 and 8). The genes lists featuring each pathway were retrieved from MSigDB and are shown in Supplementary Table 2. This analysis suggests that JNK pathway is down-regulated in $Pten^{del}$ DP cluster. It would be interesting to confirm this initial observation by additional investigations. Indeed, as JNK pathway is known to be involved in the process of negative selection of thymocytes [1], its down-regulation may impair thymocytes apoptosis.

1. Bommhardt U, Scheuring Y, Bickel C, Zamoyska R, Hünig T. MEK Activity Regulates Negative Selection of Immature CD4+CD8+ Thymocytes. *The Journal of Immunology* (2000) 164:2326–2337. doi:10.4049/jimmunol.164.5.2326

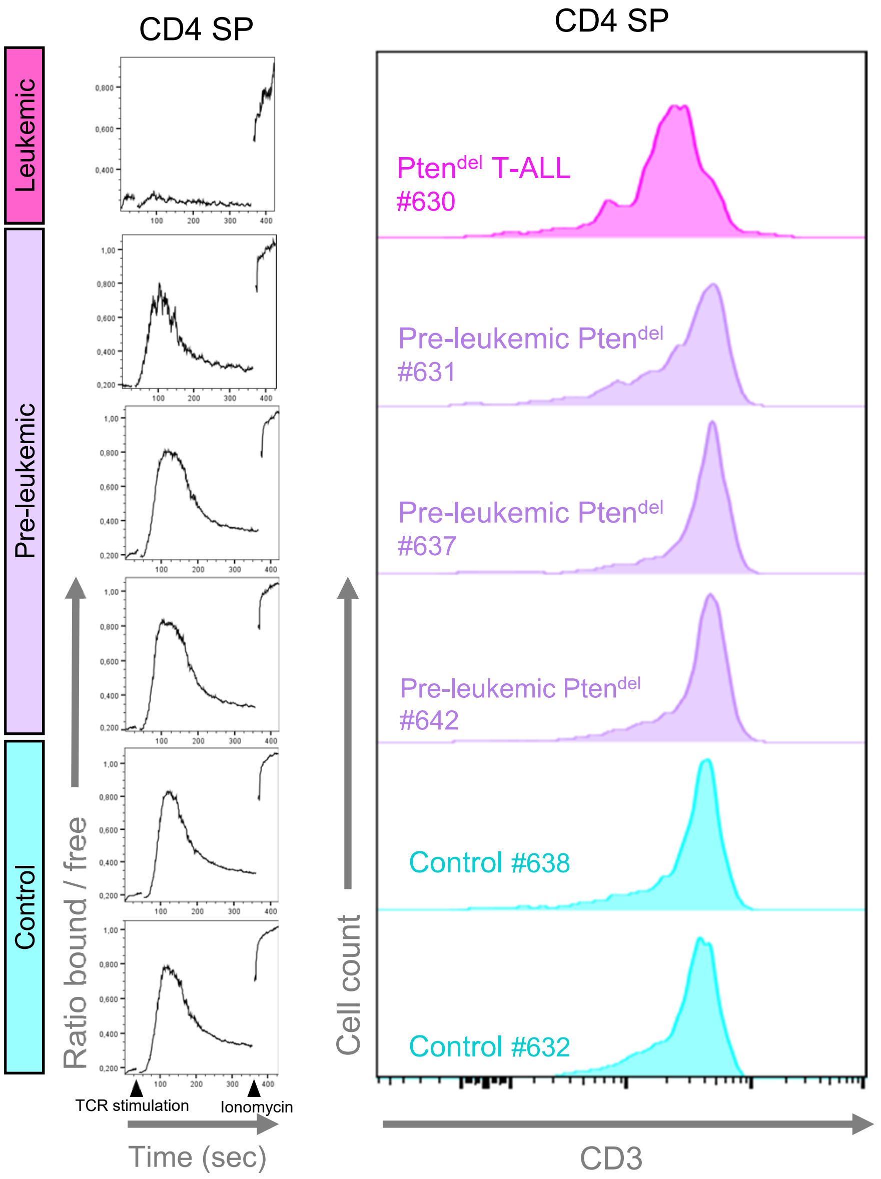

Supplementary Figure 6. Calcium flux is not impaired in non leukemic Pten^{del} thymocytes

Flow cytometry analysis of calcium flux was performed as described in Figure 2. The left panels display the assays performed with thymocytes from control mice (aged 7 and 8 weeks), pre-leukemic (aged 7 and 8 weeks) and leukemic (aged 8 weeks) Pten^{del} mice. The right panel displays flow cytometry histograms showing CD3 expression in CD4 SP thymocytes. The identification of analyzed mice is indicated (#number).

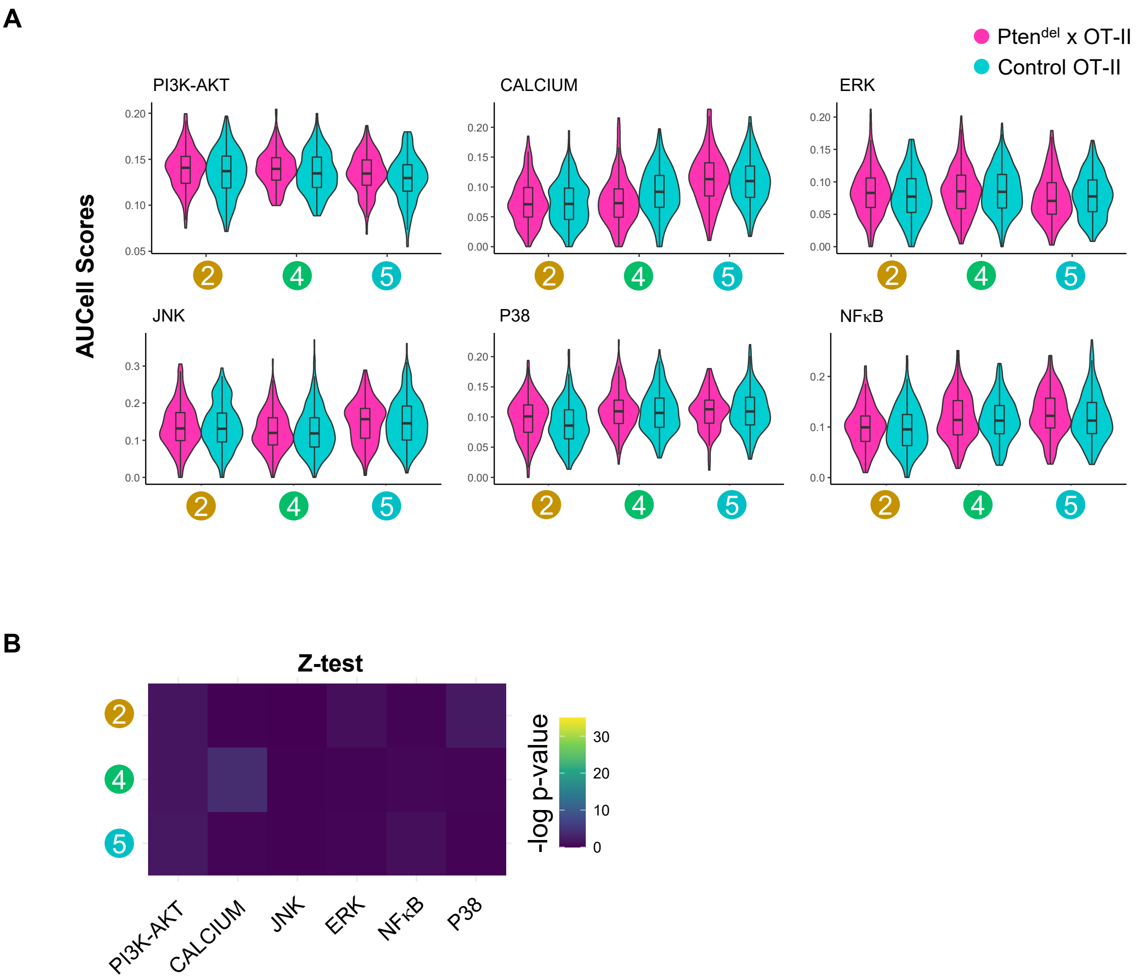

Supplementary Figure 7. Analysis of pathways activity in non-tumor cell clusters

Pathways analysis were performed as described in Figure 1. (**A**) Violin plots reporting AUCell scores for PI3K-AKT, calcium, ERK, JNK, P38 and NF-kB pathways activities in Control OT-II (blue violins) and $Pten^{del}$×OT-II (pink violins) cells of clusters 2, 4, and 5 (depicted in Figure 1C). (**B**) Heatmap of –log p-value calculated by the z-test of pathway activity, for Control OT-II *versus* $Pten^{del}$×OT-II cells within the 3 clusters indicated on the left. The analyzed pathways are indicated at the bottom of each column. Color scale is identical to Figure 1F.

```
P70227|ITPR3_MOUSE    436  VSEIRDLDFANDASSMLASAVEKLNEGFISQNDRRFVIQLLEDLVFFVSDVPNNGQNVLD  495
Q9Z329|ITPR2_MOUSE    436  LSEVRDLDFANDANKVLATTVKKLENGSITQNERRFVTKLLEDLIFFVADVTNNGQDVLD  495
P11881|ITPR1_MOUSE    437  PAEVRDLDFANDASKVLGSIAGKLEKGTITQNERRSVTKLLEDLVYFVTGGTNSGQDVLE  495
                            :*:*********..:*.: . **::* *:**:** * :*****::**:.  *.**:**:

P70227|ITPR3_MOUSE    496  IMVTKPNRERQKLMREQNILKQIFGILKAPFRDKGGEGPLVRLEELSDQKNAPYQYMFRL  555
Q9Z329|ITPR2_MOUSE    496  VVITKPNRERQKLMREQNILAQVFGILKAPFKEKAGEGSMLRLEDLGDQRYAPYKYVLRL  555
P11881|ITPR1_MOUSE    497  VVFSKPNRERQKLMREQNILKQIFKLLQAPFTD-CGDGPMLRLEELGDQRHAPFRHICRL  555
                           ::.:**************** *:* :*:*** :  *:* ::***:*.**: **:::: **

P70227|ITPR3_MOUSE    556  CYRVLRHSQEDYRKNQEHIAKQFGMMQSQIGY  587
Q9Z329|ITPR2_MOUSE    556  CYRVLRHSQQDYRKNQEYIAKNFCVMQSQIGY  587
P11881|ITPR1_MOUSE    556  CYRVLRHSQQDYRKNQEYIAKQFGFMQKQIGY  587
                           *********:*******:***:* .**.****

          Identity with ITPR3           Similarity with ITPR3
ITPR2     72.4% (110/152)               90.0% (137/152)
ITPR1     67.1% (102/152)               85.5% (130/152)
```

Supplementary Figure 8. Protein sequence comparison of PTEN-binding region across IP3 receptors

The PTEN-binding region in ITPR3 was defined by Kuchay et al. (2017); it comprises 152 amino acids (AA) ranging from AA436 to AA587. This amino acid sequence from ITPR3 was compared with the corresponding sequence from ITPR1 and ITPR2 using CLUSTAL Omega V. 1.2.4 [1]. The asterisk (*) indicates positions which have a single, fully conserved residue. The colon (:) indicates conservation between groups of strongly similar properties. The period (.) indicates conservation between groups of weakly similar properties. Percentages of identities and similarities with ITPR3 are indicated.

1. Sievers F, Wilm A, Dineen D, Gibson TJ, Karplus K, Li W, Lopez R, McWilliam H, Remmert M, Söding J, et al. Fast, scalable generation of high-quality protein multiple sequence alignments using Clustal Omega. *Mol Syst Biol* (2011) 7:539. doi:10.1038/msb.2011.75

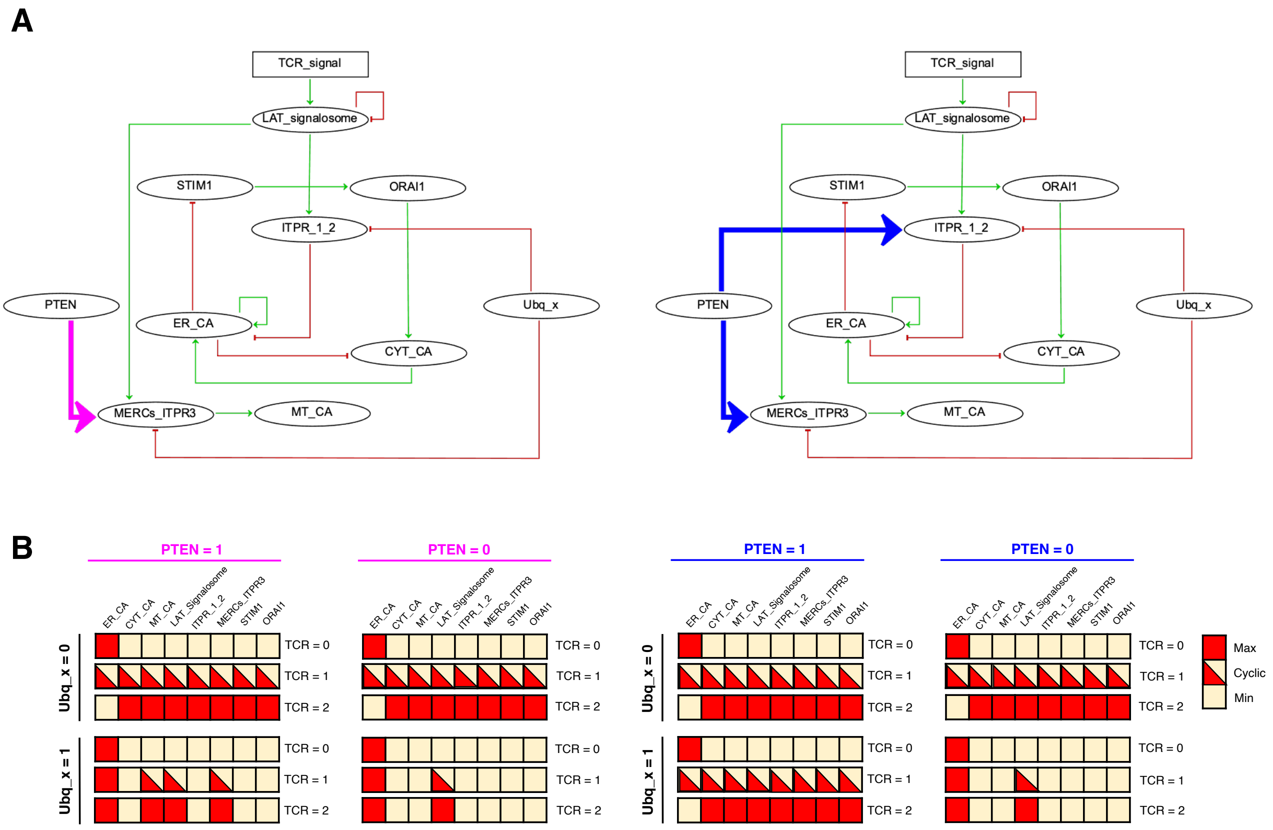

Supplementary Figure 9. Mathematical models assessing the putative role of PTEN in TCR-induced calcium flux in thymocytes

(A) Two regulatory networks representing TCR-induced calcium flux with (left) and without (right) the activation of ITPR_1_2 by PTEN. Nodes of the network represent calcium signalling component (ellipse for Boolean node, rectangular for multilevel node). The role of PTEN in each model was labelled in pink (without activation of PTEN on ITPR_1_2) and blue (with activation of PTEN on ITPR_1_2). **(B)** Attractors of the logical model for each combination of inputs: PTEN = 1 (left) and PTEN = 0 (right), Ubq_x = 0 (top) and Ubq_x = 1 (bottom), according to the 3 values of the TCR input (0/1/2). The color of each square represents the activity levels, red for active (1), yellow inactive (0). Bicolored case represents an oscillating node (cyclical attractor). The left panel shows the attractors from the model without the activation of PTEN on ITPR_1_2 and the right panel shows the attractor from model with the activation of PTEN on ITPR_1_2. A comparison of these simulations with the corresponding experimental data validates the presence of the activation of PTEN on ITPR_1_2.

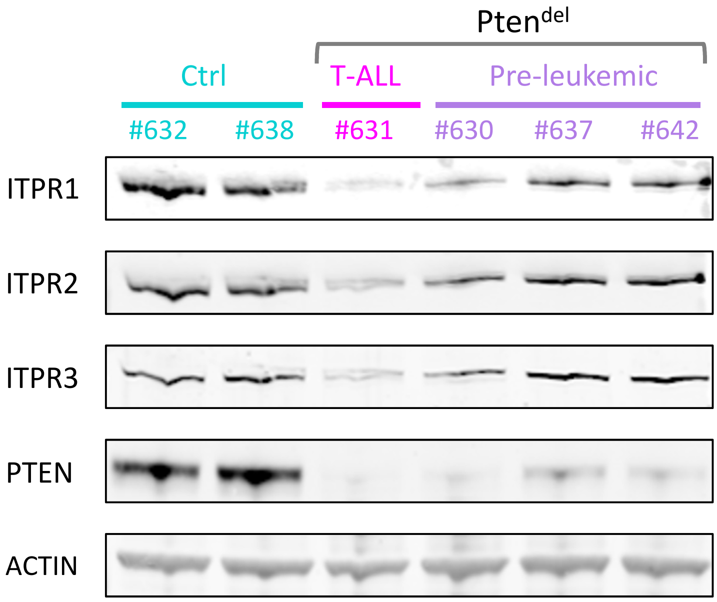

Supplementary Figure 10. Analysis of ITPRs protein expression levels in pre-leukemic Pten^{del} thymocytes

Immunoblotting assays were performed with antibodies specific for ITPR1, ITPR2, ITPR3, PTEN and ACTIN as a loading control. Thymic cells from Control (Ctrl), pre-leukemic and leukemic Pten^{del} mice were analyzed. The identification of analyzed mice (same mice as used in Supplementary Figure 6) is indicated (#number). According to our definition in Supplementary Figure 1, mice #637 and #642 were considered as 'pre-leukemic early' (normal thymus), while #630 as 'pre-leukemic late' (abnormal thymus).

Supplementary Tables

Supplementary Table 1. Antibodies used for Flow Cytometry analysis

Antibody	Company	Catalog number
**CD3e biotin**	BD Pharmingen	553059
**CD3 APC Cy7**	BD Pharmingen	560590
**CD4 V450**	BD Horizon	560468
**CD4 APC**	BD Pharmingen	553051
**CD8a PE**	BD Pharmingen	553033
**CD8a PE Cy7**	BD Pharmingen	561097
**TCR αβ PE**	BD Pharmingen	553172

Supplementary Table 2. Gene lists of pathways of interest obtained from MSigDB database

Pathways	Curator	MSigDB R pathway name	Gene list
**Calcium**	The Pathway Interaction Database (PID)	PID_TCR_CALCIUM_PATHWAY	Akap5, Batf3, Cabin1, Cd40lg, Chp1, Csf2, Fasl, Fkbp1a, Fos, Fosl1, Ifng, Il2, Il2ra, Il4, Jun, Junb, Nfatc1, Nfatc2, Nfatc3, Pou2f1, Ppp3ca, Ppp3cb, Ppp3r1, Prkaca, Ptgs2, Rcan1, Rcan2
**PI3K/AKT**	MSigDB Hallmark Gene Sets	HALLMARK_PI3K_AKT_MTOR_SIGNALING	Acaca, Actr2, Actr3, Akt1, Akt1s1, Ap2m1, Arf1, Arhgdia, Arpc3, Atf1, Cab39, Cab39l, Calr, Camk4, Cdk1, Cdk2, Cdk4, Cdkn1a, Cdkn1b, Cfl1, Cltc, Csnk2b, Cxcr4, Dapp1, Ddit3, Dusp3, E2f1, Ecsit, Egfr, Eif4e, Fasl, Fgf17, Fgf22, Gna14, Gngt1, Grb2, Grk2, Gsk3b, Hras, Hsp90b1, Il2rg, Il4, Irak4, Itpr2, Lck, Map2k3, Map2k6, Map3k7, Mapk1, Mapk10, Mapk8, Mapk9, Mapkap1, Mknk1, Mknk2, Myd88, Nck1, Nfkbib, Nod1, Pak4, Pdk1, Pfn1, Pik3r3, Pikfyve, Pin1, Pitx2, Pla2g12a, Plcb1, Plcg1, Ppp1ca, Ppp2r1b, Prkaa2, Prkag1, Prkar2a, Prkcb, Pten, Ptpn11, Rac1, Raf1, Ralb, Ripk1, Rit1, Rps6ka1, Rps6ka3, Rptor, Sfn, Sla, Slc2a1, Smad2, Sqstm1, Stat2, Tbk1, Them4, Tiam1, Tnfrsf1a, Traf2, Trib3, Tsc2, Ube2d3, Ube2n, Vav3, Ywhab
**JNK**	The Pathway Interaction Database (PID)	PID_TCR_JNK_PATHWAY	Crk, Crkl, Dbnl, Grap2, Jun, Lat, Lcp2, Map2k4, Map3k1, Map3k7, Map3k8, Map4k1, Mapk8, Prkcb
**ERK**	REACTOME	REACTOME_SIGNALLING_TO_ERKS	Braf, Crk, Crkl, Frs2, Grb2, Hras, Kidins220, Kras, Map2k1, Map2k2, Mapk1, Mapk11, Mapk12, Mapk13, Mapk14, Mapk3, Mapkapk2, Mapkapk3", "Nras, Rala, Ralb, Ralgds, Rap1a, Rapgef1, Rit1, Shc1, Sos1, Src, Ywhab
**P38**	The Pathway Interaction Database (PID)	PID_P38_ALPHA_BETA_PATHWAY	Blk, Ccm2, Cdc42, Dusp1, Dusp10, Dusp16, Dusp8, Fgr, Fyn, Hck, Lck, Lyn, Map2k3, Map2k4, Map2k6, Map3k12, Map3k3, Mapk11, Mapk14, Pak1, Pak2, Rac1, Rala, Ralb, Ripk1, Src, Tab1, Traf6, Yes1
**NFkB**	The Pathway Interaction Database (PID)	PID_NFKAPPAB_CANONICAL_PATHWAY	Atm, Bcl10, Birc2, Chuk, Cyld, Erc1, Ikbkb, Ikbkg, Malt1, Nfkb1, Nfkbia, Nod2, Prkca, Ran, Rela, Ripk2, Tnf, Tnfaip3, Tnfrsf1a, Traf6, Ube2d3, Xpo1

Supplementary Table 3. Antibodies used for immunoblotting assays

Antibody	Company	Catalog number
**ACTIN Antibody (I-19)**	Santa Cruz	sc-1616
**PTEN Antibody**	Ozyme	9559S
**ITPR1 Antibody**	Biolegend	817701
**ITPR2 Antibody**	BIO-TECHNE (Novus Biologicals)	NB100-2466
**ITPR3 Antibody**	BD transduction	610312
**Phospho-NFAT1 (Ser54) Antibody**	Invitrogen	44-944G
**Phospho-PLCγ1 (Tyr783) Antibody**	Cell signaling	14008S
**Phospho-Akt (Ser473) Antibody**	Cell signaling	9271S
**Goat Anti-Rabbit IgG (H+L), Highly Cross-Adsorbed**	Biotium	20078
**Donkey Anti-Goat IgG (H+L), Highly Cross-Adsorbed**	Biotium	20277
**Goat Anti-Mouse IgG (H+L), Highly Cross-Adsorbed**	Biotium	20065

Supplementary Table 4. Oligonucleotide sequences

RT-qPCR Primers	Target	Sequence
**Q-Abl-S**	Mouse	tgtggccagtggagataacactc
**Q-Abl-AS**	Mouse	ttcacaccattccccattgtg
**Q-Itpr1-S**	Mouse	gggccaacagcactacagat
**Q-Itpr1-AS**	Mouse	ggatgacggtccccaacaat
**Q-Itpr2-S**	Mouse	agctgagaagcgagggtgat
**Q-Itpr2-AS**	Mouse	tcgtccgaaggaaaatgtgc
**Q-Itpr3-S**	Mouse	gctcttcatccagcccttc
**Q-Itpr3-AS**	Mouse	agctggtgttgcagttgaca

Supplementary Table 5. Logical equation of each component in calcium model

Component	Logical Equation
**TCR_signal**	input {0,1,2}
**PTEN**	input {0,1}
**Ubq_x**	Input {0,1}
**LAT_signalosome**	TCR_signal:2<br>TCR_signal:1 & !LAT_signalosome
**ER_CA**	(ER_CA | CYT_CA) & !ITPR_1_2
**CYT_CA**	!ER_CA & ORAI1
**MT_CA**	MERCs_ITPR3
**ITPR_1_2**	LAT_signalosome & (PTEN | !Ubq_x)
**MERCs_ITPR3**	LAT_signalosome & (PTEN | !Ubq_x)
**STIM1**	!ER_CA
**ORAI1**	STIM1
